# High-Salt Intake Ameliorates Hyperglycemia and Insulin Resistance in WBN/Kob-*Lepr^fa/fa^* Rats: A New Model of Type 2 Diabetes Mellitus

**DOI:** 10.1155/2018/3671892

**Published:** 2018-03-20

**Authors:** Yoshiichi Takagi, Taichi Sugimoto, Masaya Kobayashi, Mitsuyuki Shirai, Fumitoshi Asai

**Affiliations:** Laboratory of Veterinary Pharmacology, School of Veterinary Medicine, Azabu University, Kanagawa, Japan

## Abstract

High-salt intake is a major risk factor for developing hypertension in type 2 diabetes mellitus, but its effects on glucose homeostasis are controversial. We previously found that high-salt intake induces severe hypertension in WBN/Kob diabetic fatty (WBKDF) rats. In the present study, we examined the effects of a high-salt intake on glucose homeostasis in WBKDF rats. Male WBKDF rats and age-matched Wistar rats at 6 weeks of age were each divided into two groups and fed either a normal-sodium (NS, 0.26%) diet or high-sodium (HS, 8%) diet for 7 weeks. Systolic blood pressure and urine volume were increased in WBKDF-HS and Wistar-HS. Body weight gain and food consumption were comparable between NS and HS in both strains. Plasma and urine glucose levels were significantly increased in WBKDF-NS but not in WBKDF-HS. HOMA-IR in WBKDF-HS was significantly lower compared with that in WBKDF-NS. The high plasma adiponectin level in WBKDF-NS compared with that in Wistar-NS was further enhanced in WBKDF-HS. Glycogen deposits and fat droplets in the livers of WBKDF-HS were reduced compared with those of WBKDF-NS. The present study demonstrated that HS intake ameliorated hyperglycemia and insulin resistance in WBKDF rats, which may be due to increased plasma levels of adiponectin.

## 1. Introduction

Metabolic syndrome is a clustering of several metabolic parameters, including hypertension, diabetes, dyslipidemia, and abdominal obesity. The incidence of metabolic syndrome is increasing rapidly in developed and developing countries [1]. Hypertension is one of the most challenging health problems leading to cardiovascular mortality and morbidity when associated with type 2 diabetes mellitus (T2DM), dyslipidemia, and obesity [2]. Dietary approaches for controlling high blood pressure have historically focused on sodium [3]. Thus, many guidelines recommend that patients with T2DM reduce their sodium intake. However, the connection between sodium intake and glucose homeostasis remains elusive.

Animal models are a useful substitute for humans in studies for both etiology and therapeutic interventions. The WBN/Kob-*Lepr^fa^* diabetic and fatty (WBKDF), a new rat strain, was developed by introducing the *fa* allele of the leptin receptor from the Zucker fatty rat into the parental WBN/Kob rat genome [4]. Compared with the parental strains, WBKDF rats are characterized by earlier-onset diabetes, more severe pancreatic complications, and endogenous insulin resistance [5–8].

Our previous study demonstrated that WBKDF-HS rats developed salt-sensitive hypertension associated with vascular dysfunction and increased oxidative stress [9]. To further validate WBKDF rats as a metabolic syndrome model, in the present study, we examined the effects of high-salt intake on glucose homeostasis in WBKDF rats in comparison with age-matched Wistar rats.

## 2. Material and Methods

### 2.1. Animals

This study was conducted in compliance with the principles of laboratory animal care and was approved by the Committee on the Ethics of Animal Experiments of Azabu University (Sagamihara, Japan). Five-week-old male WBKDF rats and age-matched male Wistar rats were obtained from Japan SLC (Hamamatsu, Japan). Animals were caged alone to allow for individual measurements of food consumption. All rats were maintained under stringent environmental conditions that included strict adherence to temperature (21 ± 1°C), humidity (55 ± 5%), and lighting (illuminated from 07:00 to 19:00).

### 2.2. Research Protocol

WBKDF rats (*n* = 16) and Wistar rats (*n* = 16) at 6 weeks of age were allocated into one of 2 groups (*n* = 8 in each group) and given either the normal-sodium diet (NS, NaCl content 0.26%) or the high-sodium diet (HS, NaCl content 8%) for 7 weeks: Wistar rats on the NS (Wistar-NS); Wistar rats on the HSD (Wistar-HS); WBKDF rats on the NSD (WBKDF-NS); and WBKDF rats on the HSD (WBKDF-HS). Animals were fed food and tap water ad libitum.

Blood samples using heparin sodium (Mitsubishi Tanabe Pharma, Tokyo, Japan) as an anticoagulant were collected from the tail vein of nonfasted and conscious rats at 6, 10, and 13 weeks of age. Urine was collected for 24 h from rats at 6, 9, and 13 weeks of age, while the animals were kept individually in metabolic cages to measure the urine volume and the urine glucose concentration. Urinary samples were collected by placing the rats at 6, 9, and 13 weeks of age in metabolic cages with their respective diets and water for 24 h. After the urine volume had been measured, the glucose and sodium concentrations in the urine were calculated.

When the rats were 13 weeks old, we performed the intravenous glucose tolerance test (IVGTT), after which the rats were euthanized by exsanguination under anesthesia with pentobarbital sodium (60 mg/kg IP; Kyoritsu Seiyaku, Tokyo, Japan). At the end of the study, the pancreas, liver, mesenteric fat pad, and epididymal fat pad were removed and weighed.

### 2.3. Measurement of Physiological Parameters

The body weights of the rats and their food intakes were measured once weekly at 6–13 weeks of age between 10:00 and 14:00. Systolic blood pressure (SBP) and heart rate in the conscious nonfasted state were measured at 6, 8, 10, and 12 weeks of ages using a tail-cuff blood pressure analyzer (BP98A-L, Softron, Tokyo, Japan). The rats were prewarmed for 15–20 min at 32°C to improve the detection of pulsation of the tail artery. The arithmetic mean of three successive measurements was used for SBP.

### 2.4. Intravenous Glucose Tolerance Test (IVGTT)

The IVGTT was conducted when the rats were 13 weeks old. After fasting for 18 h, the rats were anesthetized with pentobarbital sodium (50 to 60 mg/kg IP). Glucose (20% *w*/*v*; Otsuka Pharmaceutical, Tokyo, Japan) was injected into the femoral vein at a dose of 0.5 g/kg. Equivalent volumes (0.2 mL) of blood were sampled from the jugular vein at 0, 2, 5, 10, and 20 min. Heparinized sodium was used as an anticoagulant, and plasma was separated by centrifugation (2000 ×g for 15 min). The plasma levels of glucose and insulin were then analyzed. The area under the curves (AUCs) for plasma glucose and insulin, which represented the total glucose level and total insulin secretion during IVGTT, was calculated according to the trapezoidal rule.

Insulin resistance (IR) was assessed by the homeostasis model assessment of insulin resistance (HOMA-IR), and insulin secretion was evaluated with the *β*-cell function index of HOMA (HOMA-*β*), both of which were calculated using the fasting plasma glucose and insulin levels [10].

### 2.5. Measurement of Blood and Urine Biomarkers

Heparinized plasma was separated from the collected blood by centrifugation at 3000 ×g for 10 min. Plasma concentrations of alanine transaminase (ALT), aspartate aminotransferase (AST), total cholesterol (T-CHO), phospholipid (PL) and triglycerides (TG), and glucose in both the plasma and urine were measured using an automatic analyzer (JCA-BM 2250; JEOL Ltd., Tokyo, Japan). Plasma levels of insulin (Morinaga Institute of Biological Science Inc., Yokohama, Japan) and adiponectin (Otsuka Pharmaceutical Co. Ltd., Tokyo, Japan) were quantitated with ELISA kits.

### 2.6. Histopathological Examination

The excised pancreas and liver were immediately fixed in 10% phosphate-buffered paraformaldehyde, and the tissues were then paraffin embedded employing standard techniques and thin sectioned (3–5 *μ*m). The sections of the pancreas were immunohistochemically examined for insulin (A0504, Dako Japan, Kyoto, Japan). The sections of the liver were stained with hematoxylin and eosin (HE), periodic acid-Schiff (PAS), and Oil Red O.

### 2.7. Statistical Analysis

Data are presented as means ± standard error of the mean (SEM). Significant differences between the mean values were tested by two-way ANOVA followed by post hoc Tukey tests. *P* values less than 0.05 were considered to indicate significance. The data were analyzed using Graph Pad Prism version 7.0. (GraphPad software, San Diego, CA, USA).

## 3. Results

### 3.1. Body Weights and Food Consumption

**Figure 1:**
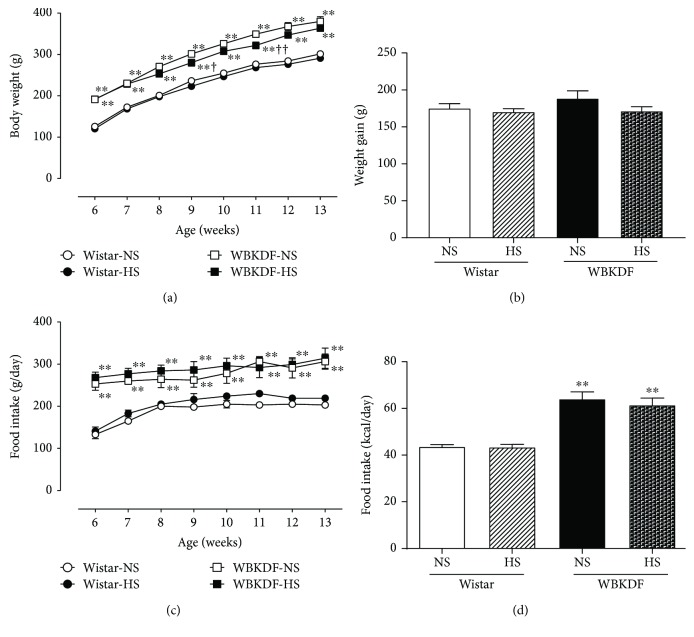
Effects of high-salt intake on body weight (a), food intake (g/day) (b), and food intake (kcal/day) (c) in WBKDF and Wistar rats. Data are expressed as mean ± SE (*n* = 8). ^∗∗^*P* < 0.01 versus Wistar rats on the same diet; ^†^*P* < 0.05 versus the same strain of rat on the NS diet; ^††^*P* < 0.01 versus the same strain of rat on the NS diet.

The body weights of WBKDF-NS rats were higher than those of Wistar-NS rats throughout the study (Figure 1(a)). There were no significant differences in body weight or body weight gain among the four groups at the end of the study (Figures 1(a) and 1(b)). At 6 weeks of age, the food intake by the WBKDF rats was two times higher than that by the Wistar rats (Figures 1(c) and 1(d)), confirming that WBKDF rats with the *fa* gene had hyperphagia. During food manipulation, both groups of WBKDF rats consumed more food and calories than Wistar rats (Figures 1(c) and 1(d)), but there was no significant difference between the NS and HS groups.

### 3.2. Organ Weight

Cardiometabolic parameters in rats from all four groups at the end of the experimental period are shown in Table 1. The weights of the epididymis and mesentery fat pads in WBKDF-NS were significantly higher than those in Wistar-NS. HS loading caused significant reduction in the weights of the epididymis (*P* < 0.05) and mesentery (*P* < 0.01) fat tissues in Wistar rats but not in WBKDF rats.

The liver weight in WBKDF-NS was significantly higher than that in Wistar rats. Inversely, the pancreas weight in WBKDF-NS was significantly lower compared with that in Wistar-NS. HS loading caused a significant reduction in the liver weight but not in the pancreas weight in WBKDF rats (Table 1).

### 3.3. Blood Pressure

Neither Wistar-NS nor WBKDF-NS exhibited significant changes in SBP throughout the experiment, and there were no significant differences in SBP between Wistar-NS and WBKDF-NS (Table 1). In contrast, both WBKDF-HS and Wistar-HS demonstrated significant elevation of SBP, which was more prominent (*P* < 0.01) in WBKDF-HS than in Wistar-HS (Table 1).

### 3.4. Blood Chemistry

WBKDF-NS rats had significantly higher levels of plasma ALT, TG, T-CHO, and PL compared with Wistar-NS rats. Plasma levels of ALT and AST in WBKDF-HS were significantly lower than those in WBKDF-NS, but there was no difference in plasma levels of TG, T-CHO, or PL between WBKDF-NS and WBKDF-HS (Table 1).

Plasma levels of adiponectin in WBKDF-NS rats were significantly higher than those in Wistar-NS rats at 13 weeks of age (*P* < 0.01). Both HS groups had higher plasma levels of adiponectin compared with NS groups, and only the differences in WBKDF rats were significant (*P* < 0.01, Table 1).

### 3.5. Nonfasting Plasma Levels of Glucose and Insulin

The nonfasting plasma glucose levels in the Wistar groups remained constant during the experimental period (data not shown). In contrast, WBKDF-NS rats developed hyperglycemia, and their plasma glucose levels at 13 weeks of age were significantly (*P* < 0.01) higher than those in Wistar rats. The HS diet inhibited the increase in plasma glucose level in WBKDF-NS rats (Table 1).

Nonfasting plasma insulin levels in WBKDF-NS rats were significantly higher than those in Wistar-NS rats at 13 weeks of age. However, there was no significant difference in plasma insulin between NS groups and HS groups of both strains (Table 1).

### 3.6. Intravenous Glucose Tolerance Test (IVGTT)

**Figure 2:**
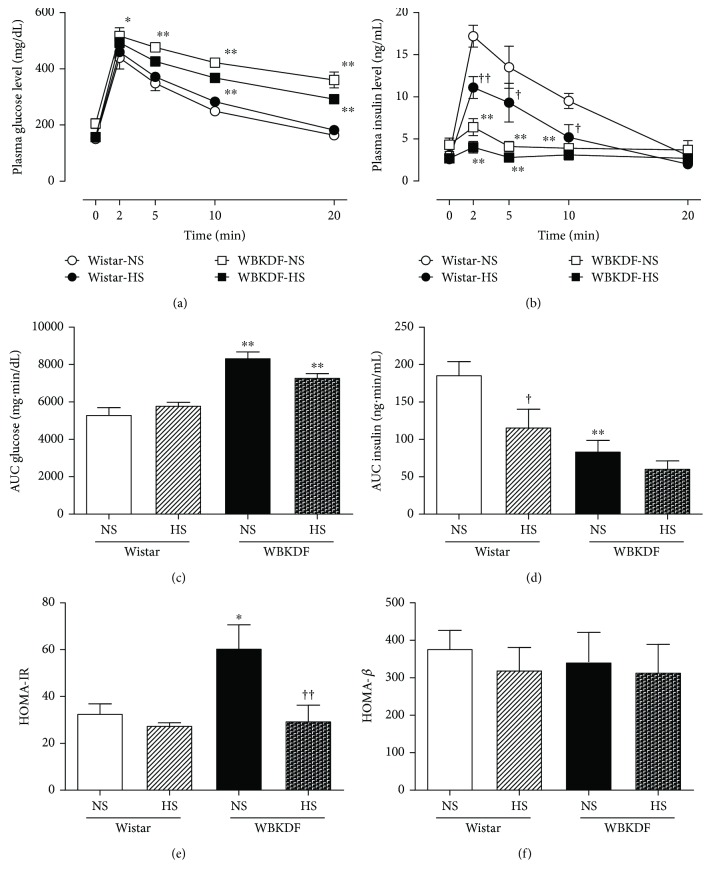
Effects of high-salt intake on plasma glucose concentration (a), plasma insulin concentration (b), AUC-glucose (c), AUC-insulin (d), and HOMA-IR (e) during the intravenous glucose tolerance test (IVGTT) in WBKDF and Wistar rats. Data are expressed as mean ± SE (*n* = 8). ^∗^*P* < 0.05 versus Wistar rats on the same diet; ^∗∗^*P* < 0.01 versus Wistar rats on the same diet; ^†^*P* < 0.05 versus the same strain of rat on the NS diet; ^††^*P* < 0.01 versus the same strain of rat on the NS diet.

The IVGTT was performed on anesthetized WBKDF and Wistar rats at 13 weeks of age after 18 h fasting. There was no significant difference in the fasted plasma glucose levels before glucose loading among all groups of rats (Figure 2(a)). Significantly (*P* < 0.01) higher plasma glucose levels in WBKDF-NS rats compared with that in Wistar-NS were observed at 2, 5, 10, and 20 min after glucose loading. Compared with WBKDF-NS, WBKDF-HS exhibited lower plasma glucose levels after glucose loading, but the difference was not significant (Figure 2(a)).

No significant differences in fasted plasma insulin levels were observed among the four groups before glucose loading (Figure 2(b)). The plasma insulin levels in WBKDF-NS rats at 2, 5, and 10 min after glucose loading were significantly (*P* < 0.01, Figure 2(b)) lower than those in Wistar-NS rats. Plasma insulin was lower in HS groups when compared with the respective NS groups, but the difference was only significant for Wistar rats (Figure 2(b)).

The plasma glucose and insulin levels measured periodically during IVGTT were used to calculate AUCs as indexes of glucose intolerance and insulin secretion, respectively. The glucose AUC value for the WBKDF-NS group was significantly higher than that for the Wistar group (*P* < 0.01; Figure 2(c)). The glucose AUC value for WBKDF-HS was lower than that for WBKDF-NS (*P* = 0.053; Figure 2(c)). The insulin AUC value for WBKDF-NS was significantly lower than that for Wistar-NS (*P* < 0.01; Figure 2(d)). The insulin AUC values for the HS groups were lower than those for the NS groups, but only values for Wistar rats were significantly different (*P* < 0.05, Figure 2(d)).

HOMA-IR was significantly (*P* < 0.05) higher in WBKDF-NS compared with that in Wistar-NS (Figure 2(e)). HS had no significant effects on HOMA-IR in Wistar rats, but HS significantly (*P* < 0.01) reduced HOMA-IR in WBKDF rats (Figure 2(e)). In contrast, there were no significant differences in HOMA-*β* among the four groups (Figure 2(f)).

### 3.7. Urine Volume and Concentration of Glucose and Sodium

The urine volume in WBKDF-NS rats was significantly higher than that in Wistar-NS rats. Urine volumes in HS groups were significantly higher than those in NS groups in both strains at 6, 9, and 13 weeks of age, whereas the urine volume in WBKDF-HS rats was higher than that in Wistar-HS rats. High urinary glucose excretion was observed in WBKDF-NS rats but not in WBKDF-HS rats (Table 1).

### 3.8. Histopathological Examination of the Pancreas and Liver

**Figure 3:**
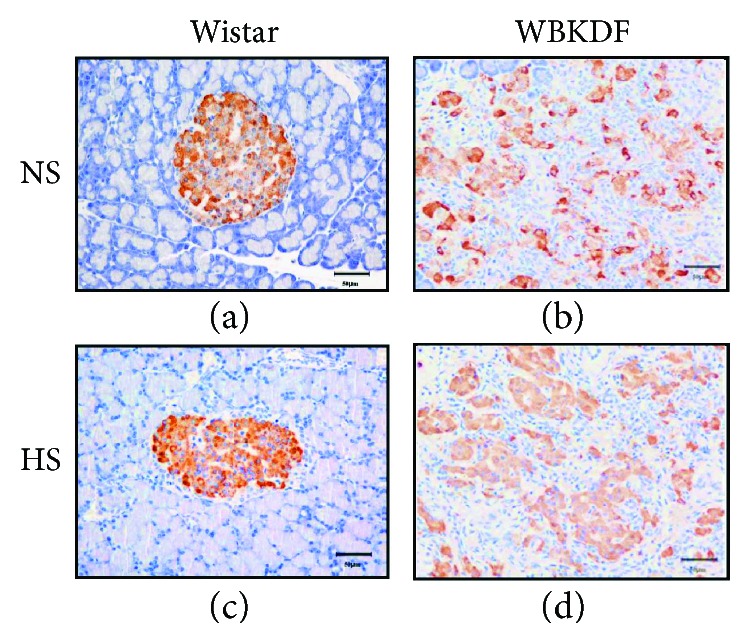
Histopathological examination of the pancreas in WBN/Kob-*Lepr^fa^* (WBKDF) and Wistar rats fed normal-sodium (NS) or high-sodium (HS) diets. Representative immunostaining of insulin in (a) Wistar-NS, (b) WBKDF-NS, (c) Wistar-HS, and (d) WBKDF-HS. Scale bars = 50 *μ*m.

Histopathology of the pancreas in WBKDF-NS rats at 13 weeks of age was characterized by loss of acinar cells, but this was not observed in Wistar-NS (Figures 3(a) and 3(b)). However, there were no morphological differences in the pancreas between the NS group and HS group in both strains (Figures 3(c) and 3(d)).

**Figure 4:**
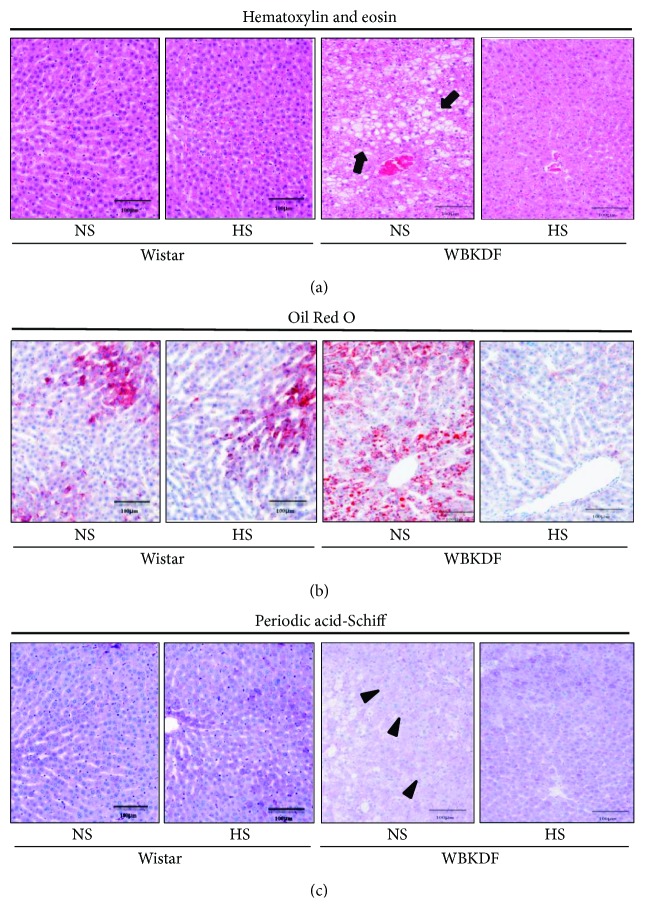
Histopathological examination of the liver in WBN/Kob-*Lepr^fa^* (WBKDF) and Wistar rats fed normal-sodium (NS) or high-sodium (HS) diets. Representative immunostaining of (a) hematoxylin and eosin (HE), (b) Oil Red O, and (c) periodic acid-Schiff (PAS) in WBKDF and Wistar rats. Scale bars = 50 *μ*m.

The histopathological analysis on the liver is shown in Figure 4. There were no morphological differences in the livers between the NS group and HS group of Wistar rats. In contrast, WBKDF-NS rats had increased hepatic lipid deposits in the peripheral periportal zone compared with WBKDF-HS rats (Figures 4(a) and 4(b)). In addition, the livers of WBKDF-NS rats had less glycogen deposition than those of WBKDF-HS rats (Figure 4(c)).

## 4. Discussion

T2DM patients are more susceptible to hypertension because of their increased salt sensitivity compared with nondiabetic individuals [11, 12]. The studies on the influence of HS intake have been limited to hypertensive patients and animals, and little is known about its influence on T2DM. The male WBKDF rat used in this study is a new metabolic syndrome model exhibiting obesity, dyslipidemia, and T2DM [4–6, 8]. Recently, we found that HS intake produces salt-sensitive hypertension in WBKDF rats, suggesting this strain as a useful animal model of metabolic syndrome [9]. This encouraged us to examine the effects of HS intake on T2DM model rats with salt sensitivity.

The main findings of this study are the following: (1) SBP and urine volume increased in WBKDF-HS and Wistar-HS. (2) Body weight gain and food consumption were comparable between NS and HS in both strains. (3) Plasma and urine glucose levels were significantly increased in WBKDF-NS, but not in WBKDF-HS rats. (4) The higher plasma adiponectin level in WBKDF-NS compared with that in Wistar-NS was further increased in WBKDF-HS rats. (5) Reduced glycogen deposits and accumulated fat droplets in the livers of WBKDF-NS rats were ameliorated in WBKDF-HS. (6) Impaired glucose tolerance and high HOMA-IR, a parameter of insulin resistance, observed in WBKDF-NS were ameliorated in WBKDF-HS rats.

According to our previous report [9], the HS diet containing 8% NaCl was demonstrated to be suitable for evaluating the long-term effects of HS in experimental animals [13]. As in the previous study [9], HS significantly increased SBP in WBKDF rats throughout the experiment. However, this salt-sensitive hypertension in WBKDF-HS was associated with the significant inhibition of hyperglycemia evident in WBKDF-NS rats. Although there are few studies examining the relationship between HS intake and hyperglycemia in T2DM model rats, our current data is consistent with reports on SDT-*Lepr^fa^* rats fed HS diets exhibiting reduction in plasma glucose levels [14].

The WBKDF rat carries the fatty mutation (*fa*) in the leptin receptor gene, and homozygous animals (*fa/fa*) exhibit hyperphagia and obesity, in addition to insulin resistance and glucose intolerance [5–8]. Although nonfasting plasma glucose levels in WBKDF-NS rats were significantly higher than those in WBKDF-HS rats, there were no differences in fasting plasma glucose levels between WBKDF-HS and WBKDF-NS rats at 13 weeks of age. Early studies [15] demonstrated that food restriction delayed the development of hyperglycemia in WBKDF rats. These results suggest that hyperglycemia in the WBKDF rat is mainly due to hyperphagia [16]. However, our present and previous studies [9] found no significant differences in food consumption. Thus, inhibition of hyperglycemia by HS intake in WBKDF rats is unlikely due to reduced food and calorie intake.

The renal sodium-glucose cotransporter 2 (SGLT2) plays a crucial role in the regulation of renal glucose transport and sodium reabsorption [17]. SGLT2 inhibitors have been used to treat T2DM through inhibition of glucose reabsorption from the proximal renal tubule [18]. Thus, we examined the urine volume and the concentration of glucose. High urine glucose levels, a typical sign of diabetes, were observed in WBKDF-NS rats but not in WBKDF-HS rats. Thus, the inhibition of hyperglycemia by HS intake in WBKDF rats was due to a different mechanism than SGLT2 inhibition.

The GLP-1 receptor agonist was previously reported to inhibit hyperglycemia via its insulinotropic action in WBKDF rats [16]. In contrast, the normoglycemic WBKDF-HS group had lower insulin concentrations than the hyperglycemic WBKDF-NS group, but there were no significant differences in pancreatic *β*-cell injury between both groups. HS intake leads to a significant reduction in fasting plasma levels of glucose and insulin in human patients [19]. As the plasma insulin AUCs in the WBKDF-NS group was significantly lower than that in the Wistar-NS group, the reduced insulin secretory function in the WBKDF rats may be due to pancreatic *β*-cell dysfunction associated with chronic pancreatitis. Furthermore, both plasma insulin levels and HOMA-*β* were comparable between NS and HS groups in both strains of rats. Thus, the ameliorating effects of HS on hyperglycemia and glucose intolerance in WBKDF rats are unlikely due to the increase in plasma insulin levels.

Our previous and present studies have demonstrated that WBKDF rats have high HOMA-IR values, an indicator of insulin resistance, which are the hallmark of T2DM. Moreover, the IVGTT in the current study revealed that HS intake caused a marked improvement in insulin resistance in WBKDF rats. Obesity and insulin resistance are closely related with fatty liver [20]. Obesity-induced fat accumulation in adipose tissues and the liver, increased fatty acid synthesis in the liver and increased TG level in the plasma are associated with insulin resistance [20]. Indeed, the livers of WBKDF-NS rats had fat accumulation and reduced glycogen due to insulin resistance. The ameliorated histological hepatic changes observed in WBKDF-NS rats due to HS intake may be associated with insulin resistance in the liver. These results suggest that HS intake inhibited hyperglycemia by ameliorating insulin resistance in WBKDF rats.

In contrast to WBKDF rats, there was no obvious change in insulin sensitivity between Wistar-NS and Wistar-HS rats. The connection between sodium intake and insulin resistance has been debated [21]. Our finding on insulin resistance conflicts with the observation that the HS intake led to insulin resistance in rats [22–24]. The reason for this apparent discrepancy among the rat studies is unclear, but one possible explanation is that the differences in the rats, such as the rat strain, salt-sensitivity, and insulin-sensitivity, in addition to experimental conditions, including sodium concentration in the diet and duration of HS feeding, may be involved. Indeed, Sprague–Dawley rats, which were used in the aforementioned studies, are generally considered to be salt resistant [25].

The current study demonstrated that the HS diet increased plasma levels of adiponectin, one of the major adipocyte-secreted proteins [26], in WBKDF rats and Wistar rats. These results are in line with early studies reporting that a high-salt intake increased plasma adiponectin levels in normal human volunteers [27] and rodents [28, 29]. Previous studies clarifying and characterizing insulin-sensitizing activities, in vivo tissues, and underlying mechanisms suggested that the liver is a major target tissue of adiponectin, which exerts its insulin-sensitizing effects by binding to its receptors, leading to activation of the AMP-activated protein kinase and other unknown pathways [30]. Our results are consistent with early reports that treatment with recombinant adiponectin markedly alleviated hepatomegaly and fatty liver, as well as significantly attenuated elevated levels of serum ALT in nonalcoholic obese *ob/ob* mice [31], which spontaneously develop hyperinsulinemia, insulin resistance, and steatosis due to an inherited leptin deficiency [32]. Taken together, these results suggest that increased adiponectin by HS intake improved hyperglycemia and insulin resistance in WBKDF rats.

Our previous study demonstrated that WBKDF-HS rats developed renal damage and hypertension, which may have been associated with vascular dysfunction and increased oxidative stress [9]. Other groups have reported HS-induced renal tubular damage in spontaneously hypertensive rats [33] and increase in angiotensin II in the proximal tubular system via activation of the intrarenal renin-angiotensin system (RAS) [34]. In addition, angiotensin II is known to increase adiponectin levels [27, 28]. Therefore, it is probable that increased angiotensin II via activation of the intrarenal RAS system by HS intake contributes to salt-sensitive hypertension and enhances plasma adiponectin, which subsequently leads to reduced insulin resistance in WBKDF-HS rats. Further studies are required to examine this hypothesis.

The present study has several limitations. Firstly, disturbed regulation of adipokines, including resistin and visfatin, as well as adiponectin, is implicated in the development of insulin resistance and T2DM [35]. Thus, whether inhibition of hyperglycemia by HS intake observed in WBKDF rats was solely due to adiponectin remains to be elucidated. Secondly, plasma adiponectin levels in WBKDF-NS were higher than those in Wistar-NS rats. This result contrasts with the observation that reduction of adiponectin mainly contributes to T2DM development [26]. A possible explanation for the high adiponectin level in WBKDF-NS is a compensatory mechanism that counteracts high insulin resistance in WBKDF rats. Further studies are needed to clarify this possibility and to examine the cellular and molecular mechanisms that regulate plasma levels of adiponectin in high-salt conditions.

In conclusion, the HS intake ameliorated hyperglycemia and insulin resistance in WBKDF rats, which may be due to enhancement of plasma adiponectin levels independent of the increase in BP. The WBKDF rat may be a useful model for examining the etiology of T2DM with salt-sensitive hypertension.

## Figures and Tables

**Table 1:** Cardio-metabolic parameters in Wistar rats and WBKDF rats fed either the normal sodium (NS) diet or high sodium (HS) diet for 7 weeks.

	Wistar-NS (*N* = 8)	Wistar-HS (*N* = 8)	WBKDF-NS (*N* = 8)	WBKDF-HS (*N* = 8)	ANOVA (Strain)	ANOVA (Treatment)
Organ weight (% body weight)						
Liver weight	2.9 ± 0.1	2.9 ± 0.0	3.8 ± 0.1^∗∗^	3.2 ± 0.1^††^	*P* < 0.0001	*P* = 0.0017
Pancreas weight	0.4 ± 0.0	0.4 ± 0.0	0.2 ± 0.0^∗∗^	0.2 ± 0.0^∗∗^	*P* < 0.0001	*P* = 0.3124
Mesenteric fat weight	1.4 ± 0.1	0.9 ± 0.1^††^	2.2 ± 0.1^∗∗^	1.9 ± 0.0^∗∗^	*P* < 0.0001	*P* < 0.0001
Epididynmal fat weight	1.6 ± 0.1	1.2 ± 0.1^†^	2.9 ± 0.0^∗∗^	2.9 ± 0.1^∗∗^	*P* < 0.0001	*P* = 0.0285
Heart rate (bpm)	390.6 ± 13.3	395.8 ± 17.9	326.4 ± 5.1^∗∗^	346.3 ± 9.6^∗^	*P* < 0.0001	*P* = 0.3203
SBP (mmHg)	127.9 ± 2.2	151.2 ± 3.9^†^	120.7 ± 4.1	192.0 ± 8.2^∗∗††^	*P* = 0.0027	*P* < 0.0001
Blood						
Glucose (mg/dL)	115.1 ± 3.5	115.0 ± 4.3	344.8 ± 44.2^∗∗^	127.0 ± 8.8^††^	*P* < 0.0001	*P* < 0.0001
Insulin (ng/dL)	2.4 ± 0.3	1.2 ± 0.2	15.0 ± 1.5^∗∗^	13.2 ± 0.9^∗∗^	*P* < 0.0001	*P* = 0.2161
AST (U/L)	67.4 ± 4.0	68.8 ± 7.6	74.6 ± 21.4	50.0 ± 2.7	*P* = 0.6212	*P* = 0.3262
ALT (U/L)	36.5 ± 2.7	32.1 ± 2.4	109.6 ± 24.3^∗∗^	71.6 ± 8.6	*P* = 0.0002	*P* = 0.1145
T-CHO (mg/dL)	56.5 ± 2.6	47.5 ± 3.8	95.9 ± 5.7^∗∗^	106.6 ± 10.6^∗∗^	*P* < 0.0001	*P* = 0.8960
TG (mg/dL)	27.1 ± 4.5	26.8 ± 3.1	304.4 ± 34.7^∗∗^	315.8 ± 36.4^∗∗^	*P* < 0.0001	*P* = 0.8279
PL (mg/dL)	103.9 ± 2.4	92.9 ± 4.9	206.0 ± 7.8^∗∗^	237.6 ± 13.7^∗∗^	*P* < 0.0001	*P* = 0.2272
Adiponectin (ng/mL)	1432 ± 84	1716 ± 105	2182 ± 195^∗∗^	2687 ± 65^∗∗†^	*P* < 0.0001	*P* = 0.0033
Urine						
Volume (mL/24 h)	6.3 ± 0.5	85.1 ± 1.5^††^	23.8 ± 4.7^∗^	108.8 ± 7.5^∗∗††^	*P* < 0.0001	*P* < 0.0001
Glucose (g/24 h)	0.2 ± 0.0	0.0 ± 0.0	127.9 ± 39.5^∗∗^	0.0 ± 0.0^††^	*P* = 0.0031	*P* = 0.0031

Data are expressed as mean ± SE (*n* = 8). Heart rate and SBP are for rats at 12 weeks of ages. Other parameters are for rats at 13 weeks. ^∗^*P* < 0.05 versus Wistar rats on the same diet; ^∗∗^*P* < 0.01 versus Wistar rats on the same diet; ^†^*P* < 0.05 versus the same strain of rat on the NS diet; ^††^*P* < 0.01 versus the same strain of rat on the NS diet. SBP: systolic blood pressure, AST: aspartate aminotransferase, ALT: alanine aminotransferase, T-CHO: total cholesterol, TG: triglyceride, PL: phospholipid.
